# Plasticity of Spine Structure: Local Signaling, Translation and Cytoskeletal Reorganization

**DOI:** 10.3389/fnsyn.2018.00029

**Published:** 2018-08-29

**Authors:** Yoshihisa Nakahata, Ryohei Yasuda

**Affiliations:** Neuronal Signal Transduction, Max Planck Florida Institute for Neuroscience (MPFI), Jupiter, FL, United States

**Keywords:** dendritic spine, calcium signaling, synaptic plasticity, actin cytoskeleton, CaMKII, small GTPase, FRET FLIM, LTP

## Abstract

Dendritic spines are small protrusive structures on dendritic surfaces, and function as postsynaptic compartments for excitatory synapses. Plasticity of spine structure is associated with many forms of long-term neuronal plasticity, learning and memory. Inside these small dendritic compartments, biochemical states and protein-protein interactions are dynamically modulated by synaptic activity, leading to the regulation of protein synthesis and reorganization of cytoskeletal architecture. This in turn causes plasticity of structure and function of the spine. Technical advances in monitoring molecular behaviors in single dendritic spines have revealed that each signaling pathway is differently regulated across multiple spatiotemporal domains. The spatial pattern of signaling activity expands from a single spine to the nearby dendritic area, dendritic branch and the nucleus, regulating different cellular events at each spatial scale. Temporally, biochemical events are typically triggered by short Ca^2+^ pulses (~10–100 ms). However, these signals can then trigger activation of downstream protein cascades that can last from milliseconds to hours. Recent imaging studies provide many insights into the biochemical processes governing signaling events of molecular assemblies at different spatial localizations. Here, we highlight recent findings of signaling dynamics during synaptic plasticity and discuss their roles in long-term structural plasticity of dendritic spines.

## Introduction

The dendritic spine is a small protrusive structure that typically houses a single excitatory postsynapse. The spine is made of a head (~0.01–1 fL) and a narrow neck (~0.1 μm in diameter) that connects the head and dendritic surface. This structure spatially restricts electrical and biochemical access from the spine head to the dendritic shaft (Svoboda et al., 1996; Bloodgood and Sabatini, 2005; Gulledge et al., 2012; Yuste, 2013). This rather unusual structure is maintained by a network of actin cytoskeleton (Hotulainen and Hoogenraad, 2010; Colgan and Yasuda, 2014). The actin network also acts as a scaffold for stably positioning channels, cell adhesion proteins and sub-spine structure such as endosomes and postsynaptic densities (PSD; Spence and Soderling, 2015).

While dendritic spines can be stable for months to years (Grutzendler et al., 2002; Trachtenberg et al., 2002), which perhaps is important for stable function of neuronal circuits, structural plasticity of dendritic spines are known to be correlated with circuit plasticity during learning (Trachtenberg et al., 2002; Yang et al., 2009, 2014; Hayashi-Takagi et al., 2015; Li et al., 2017). Importantly, models of long-term synaptic plasticity such as long-term potentiation (LTP) and long-term depression (LTD) also are associated with long-term enlargement and shrinkage of dendritic spines, respectively (Matsuzaki et al., 2004; Zhou et al., 2004). These forms of plasticity, termed structural LTP (sLTP) and structural LTD (sLTD), are thus perhaps the basis of long-term circuit reorganization during learning and memory (Kasai et al., 2010). Structural plasticity of dendritic spines is associated with molecular reorganization. For example, the actin cytoskeletal mesh, which maintains the spine structure, needs to be rearranged. In addition, PSD size and the number of glutamate receptors on the spine also changes (Makino and Malinow, 2009; Bosch et al., 2014; Meyer et al., 2014).

sLTP is perhaps the most studied form of spine structural plasticity. It has been shown that plasticity has several temporal phases with distinct sensitivity to pharmacological and genetic perturbations (Matsuzaki et al., 2004; Murakoshi et al., 2011). Immediately after induction (either by electrical stimulation or glutamate uncaging), spines undergo a rapid and large volume increase. This is called the transient phase, and the exact physiological role of this phase is unknown. The volume decreases over several minutes but stabilizes at a level higher than the original volume. This is called the sustained phase, and continues for more than an hour. This phase is associated with an increase in the postsynaptic sensitivity to glutamate (Matsuzaki et al., 2004; Harvey et al., 2008; Lee et al., 2009; Murakoshi et al., 2011). Depending on the conditions, the sustained phase can be protein-synthesis dependent (Nguyen and Kandel, 1997; Kelleher et al., 2004; Tanaka et al., 2008; Govindarajan et al., 2011).

The rapid and sustained structural remodeling of spines depends crucially on intracellular signaling networks to orchestrate posttranslational modifications and nascent protein synthesis. In this review article, we highlight recent findings demonstrating intracellular and extracellular molecular interactions regulating actin cytoskeleton as a structural basis of spine remodeling as well as implications of activity-dependent local translation for long-lasting synaptic plasticity.

## Structural Regulation of Dendritic Spines

The principal architectural component of the spine is the actin cytoskeleton (Korobova and Svitkina, 2010). Long- and short-branched filamentous actin (F-actin) are connected through multiple actin-binding proteins (ABPs), forming a highly branched network (Hotulainen and Hoogenraad, 2010; Colgan and Yasuda, 2014). Therefore, dynamic remodeling of actin networks within dendritic spines is essential for activity-dependent structural changes of spines (Okamoto et al., 2004; Honkura et al., 2008; Frost et al., 2010).

**Figure 1:**
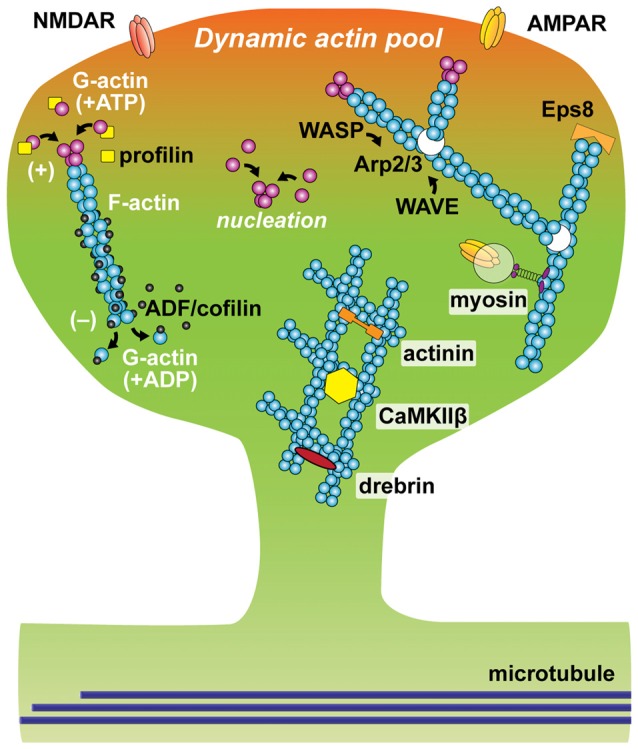
Schematic of actin and actin-binding proteins (ABPs) in a dendritic spine. Filamentous actin (F-actin) is formed by polymerization of globular actin (G-actin). The constant process of polymerization of ATP-bound G-actin (magenta oval) at barbed (plus) end and depolymerization of ADP-bound G-actin (cyan oval) at pointed (minus) end is called actin tread-milling. Profilin (yellow square) binds to monomeric G-actin and accelerates exchange of its nucleotide from ADP to ATP, as the result enhancing actin polymerization. ADF/cofilin (black oval) binds to ADP-bound actin and accelerates actin depolymerization at a low concentration. Arp2/3 complex (white oval) nucleates actin branching. The function of Arp2/3 complex is activated by Wiskott-Aldrich syndrome family protein (WASP) and inhibited by WASP family verprolin-homologous protein (WAVE). Epidermal growth factor receptor kinase substrate 8 (Eps8), binds to barbed-end and stabilizes actin filaments. Cross-linking proteins including actinin, CaMKIIβ and drebrin stabilize F-actin and form actin network. Active vesicular transport along F-actin is regulated by myosin.

F-actin is formed by polymerization of monomeric globular actin (G-actin). These two forms of actin undergo a cycle called tread-milling: ATP-bound G-actin is added to the fast-growing end (barbed end or plus end) and ADP-bound G-actin is dissociated from the other side (pointed end or minus end) of F-actin (Figure 1). The cycle of tread-milling in spines is fast: on average, most actin monomers in a filament are replaced every minute. However, it was found that a small population of actin near the base of the spine neck is much more stable (Honkura et al., 2008). This actin population remains in filaments for more than tens of minutes. The balance between actin polymerization and depolymerization plays a major role in structural plasticity of dendritic spines (Hotulainen and Hoogenraad, 2010). For example, during spine volume increases associated with sLTP, the balance is shifted toward actin polymerization, thereby elongating actin filaments and expanding the actin network (Okamoto et al., 2004; Honkura et al., 2008; Bosch et al., 2014).

Changes in the actin network are regulated by multiple ABPs (Figure 1). These proteins play roles in many different aspects of actin dynamics: actin polymerization, depolymerization, nucleation, branching, capping, cross-linking and trafficking. For example, the actin-related protein-2/3 (Arp2/3) complex nucleates the formation of actin filaments that branch off existing filaments at a specific angle (70 degrees). Thus, it is an important component for producing the mesh-like structure of actin filaments. It is activated and inhibited by members of the Wiskott-Aldrich syndrome family protein (WASP) and WASP family verprolin-homologous protein (WAVE), respectively (Soderling and Scott, 2006). Disruption of the Arp2/3 complex results in an increase in immature spines and abnormal behavior (Kim et al., 2013). Another ABP, profilin, plays an important role for actin polymerization by binding G-actin and accelerating the exchange of ADP to ATP, enhancing the rate of tread-milling (Ackermann and Matus, 2003; Neuhoff et al., 2005). At the plus-end of actin filaments, actin-capping proteins such as epidermal growth factor receptor kinase substrate 8 (Eps8), bind to and stabilize actin filaments (Menna et al., 2013). The function of Eps8 is downregulated by MAPK/ERK-dependent phosphorylation (Menna et al., 2009). Another important ABP for synaptic plasticity is ADF/cofilin (Zhou et al., 2004; Bosch et al., 2014; Rust, 2015). The action of ADF/cofilin family proteins is known to be concentration-dependent. While it induces depolymerization at the minus-end at a low concentration, ADF/cofilin can co-polymerize with actin filaments, stabilizing filamentous structure at a high concentration (Andrianantoandro and Pollard, 2006). LIM kinase (LIMK)-dependent phosphorylation inhibits its action. During LTP, ADF/cofilin shows biphasic phosphorylation: transient increase followed by decrease in the stimulated spine (Chen et al., 2007). This dynamic regulation of phosphorylation causes a persistent accumulation of ADF/cofilin at the neck of the stimulated spine (Chen et al., 2007; Bosch et al., 2014; Noguchi et al., 2016). During this process, ADF/cofilin appears to form the polymerized structure, stabilizing actin cytoskeleton for a long time (more than ~30 min). For formation and stabilization of complex actin networks, cross-linking proteins such as drebrin, α-actinin and Calcium/calmodulin-dependent protein kinase type II subunit β (CaMKIIβ) play a key role for bundling F-actin (Hotulainen and Hoogenraad, 2010; Kim et al., 2015). The transient exit and reentry of drebrin A in the spine head is thought to be important for spine remodeling (Bosch et al., 2014; Mizui et al., 2014; Shirao et al., 2017). In addition, myosin, a superfamily of ATP-driven motor proteins, regulates spine structure via its multiple functions including protein trafficking and contractile bundling of F-actin networks (Ryu et al., 2006; Correia et al., 2008; Wang Z. et al., 2008; Korobova and Svitkina, 2010). Thus, structural plasticity of dendritic spines requires spatiotemporal coordination of ABPs. While exact actin dynamics during synaptic plasticity remains elusive, it has been proposed that actin network becomes fluid in the initial phase of structural changes, perhaps due to the dissociation of ABPs, allowing actin to be reorganized, and then subsequently stabilized by the re-binding of ABPs (Okamoto et al., 2004; Kim et al., 2015).

## Transient Ca^2+^ Elevation: The Trigger of Multiple Signaling Cascades for Spine Remodeling

Since regulation of actin cytoskeleton is tightly coupled with changes in spine structure, the signaling system connecting synaptic activity and various ABPs plays an important role in spine structural plasticity. Indeed, recent studies using molecular imaging, such as FRET-based fluorescence lifetime microscopy (FLIM) imaging have revealed the spatiotemporal dynamics of key signaling molecules regulating actin cytoskeleton in dendritic spines (Nishiyama and Yasuda, 2015).

Strong excitatory synaptic inputs induce postsynaptic Ca^2+^ elevation through NMDA receptors and/or voltage-gated Ca^2+^ channels (VGCCs) in the activated spine (Sabatini et al., 2002). Ca^2+^ elevation in spines triggers signaling cascades for long-term synaptic plasticity including LTP and sLTP. Ca^2+^ binds to calmodulin (CaM), a Ca^2+^-binding protein and Ca^2+^ bound CaM (Ca^2+^/CaM) subsequently activates Ca^2+^/CaM-dependent kinases and phosphatases such as CaMKII and calcineurin (CaN; Lee et al., 2009; Fujii et al., 2013; Chang et al., 2017). Traditionally, it has been considered that CaMKII and CaN are exclusively activated during postsynaptic activation and plays roles in LTP and LTD, respectively (Malenka and Bear, 2004). Consistent with this, it has been shown that CaMKII is activated during the induction of sLTP (Lee et al., 2009; Chang et al., 2017). Also, CaN was shown to be necessary for sLTD (Zhou et al., 2004; Oh et al., 2015). However, a recent study suggested that CaN is activated in LTP-inducing stimuli, as well as LTD-inducing stimuli (Fujii et al., 2013). Furthermore, it has been reported that CaMKII activity is required for inducing LTD as well as LTP (Coultrap et al., 2014; Goodell et al., 2017). Both CaMKII and CaN activity rapidly decay over a few seconds (Fujii et al., 2013; Chang et al., 2017).

It should be worth mentioning that CaMKII activation has been proposed to act as a biochemical memory lasting more than hours (Lisman et al., 2002). Upon Ca^2+^/CaM binding, active CaMKII can undergo autophosphorylation at Thr286 (for CaMKIIα or Thr287 for CaMKIIβ), which makes the kinase activity independent of Ca^2+^/CaM binding. This Ca^2+^/CaM-independent activation, which is often referred to as “autonomous” activity, could persist for a long time after Ca^2+^ decays. Indeed, it has been found that autophosphorylation at Thr286 persists for more than ~1 h after LTP induction (Barria et al., 1997). Furthermore, transgenic mice bearing a single point mutation at Thr286 (T286A) of CaMKIIα showed deficits in LTP and learning. However, pharmacological studies showed that, while inhibition of CaMKII during the induction inhibits the induction of LTP, inhibition after the induction of LTP does not reverse LTP, suggesting that CaMKII is required for induction, but not for the maintenance, of LTP (Buard et al., 2010). Similarly, CaMKII is found to be necessary for the formation, but not for the maintenance or retrieval, of amygdala-dependent fear memory (Buard et al., 2010). More recent study with optogenetic inhibitor of CaMKII further refined the temporal window of CaMKII action (Murakoshi et al., 2017). This study showed that CaMKII activation is necessary only for the first ~1 min of LTP induction. In addition, CaMKII activation in amygdala during the training (~3 min), but not after the training, is required for fear memory formation in the inhibitory avoidance task (Murakoshi et al., 2017). This apparent inconsistency between the early biochemical studies and the results from pharmacological and optogenetic inhibition could be because autophosphorylation does not correlate with CaMKII activation under some conditions (Lengyel et al., 2004).

During sLTP, short CaMKII activation is relayed to diverse downstream signaling molecules including small GTPase proteins. The activity of these downstream signals lasts more than tens of minutes, reorganizing actin cytoskeleton over this time period (Yasuda, 2017). The process of small GTPase signaling will be discussed in the “Rapid Structural Remodeling of the Spine” section.

## Rapid Structural Remodeling of the Spine

### Intracellular Signaling Networks for Rapid Cytoskeletal Restructuring

**Figure 2:**
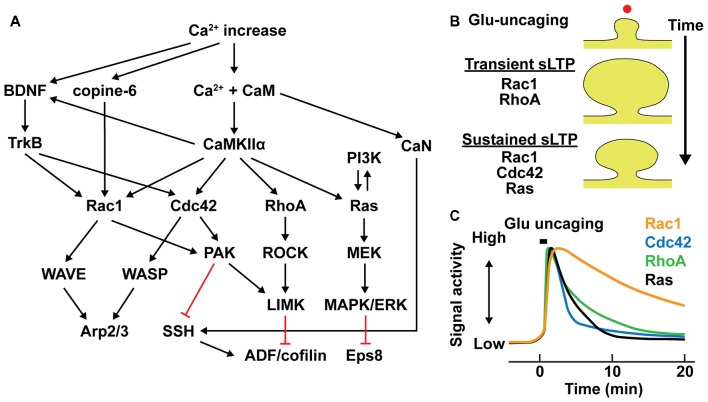
Intracellular signal regulation during structural long-term potentiation (LTP). **(A)** Signaling pathways controlling actin binding proteins (ABPs) in dendritic spines. Black arrows represent downstream activation and red lines represent downstream inhibition. **(B)** Different contributions of small GTPase activations in transient and sustained structural LTP (sLTP) in stimulated spines. Red dot represents a single spine stimulation by glutamate uncaging. **(C)** Schematic time course of small GTPase activation profiles in stimulated spines (Murakoshi et al., 2011; Oliveira and Yasuda, 2014; Hedrick et al., 2016).

Cytoskeletal remodeling during structural plasticity of spines requires activation of small GTPase proteins (Harvey et al., 2008; Murakoshi et al., 2011; Bosch et al., 2014; Hedrick et al., 2016; Figure 2A). RhoA, Cdc42, Rac1 and Ras are all activated by CaMKII, and required for sLTP (Harvey et al., 2008; Murakoshi et al., 2011; Bosch et al., 2014; Hedrick et al., 2016). In addition, knockout of Rac1 and Cdc42 from excitatory neurons causes impaired LTP and memory formation (Haditsch et al., 2009; Kim et al., 2014). RhoA activation controls spine remodeling through the activation of downstream effectors such as Rho-associated protein kinase (ROCK). Activated ROCK phosphorylates LIMK, which further phosphorylate ADF/cofilin (Arber et al., 1998). Cdc42 and Rac1 promote actin polymerization through activating WASP and WAVE, respectively. The activated WASP and WAVE bind to and upregulate Arp2/3 complex, which induces actin nucleation and thus spine enlargement (Hlushchenko et al., 2016). Cdc42 and Rac1 also stabilizes actin cytoskeleton by inhibiting ADF/cofilin-mediated actin depolymerization through downstream effectors p21-activated kinase (PAK)-LIMK pathway and the PAK-phosphatases slingshot (SSH) pathway (Zhou et al., 2012; Bosch et al., 2014). Recent studies suggest that Copine-6, a Ca^2+^-binding molecule is another upstream regulator of the Rac1-PAK-LIMK pathway (Reinhard et al., 2016; Burk et al., 2018).

**Figure 3:**
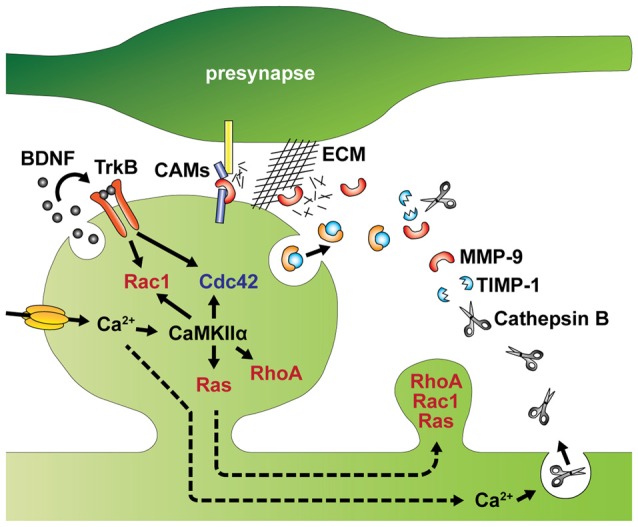
Intracellular and extracellular factors that regulate spine structural plasticity. Activity-dependent autocrine brain-derived neurotrophic factor (BDNF)-TrkB signaling activates Cdc42 and Rac1 in single spines. Ca^2+^ influx through NMDA receptors or voltage gated Ca^2+^ channels (VGCCs) activates CaMKIIα and its downstream signaling including Cdc42, Rac1, RhoA and Ras. Activation of Rac1 and Cdc42 are regulated by autocrine BDNF-TrkB signaling. Activities of RhoA, Rac1 and Ras spread to dendritic shaft and adjacent spines from stimulated spines. MMP-9 and TIMP-1 are also released from the postsynaptic cells. In addition, Ca^2+^ elevation induces lysosomal fusion with the plasma membrane, releasing Cathepsin B to outside of the cell. Extracellular Cathepsin B cleaves the tissue inhibitor of metalloproteinases-1 (TIMP-1), an endogenous inhibitor of the matrix metalloprotease 9 (MMP-9). Disinhibited MMP-9 cleaves cell adhesion molecules (CAMs) and the extracellular matrix (ECM), which facilitate structural remodeling of spines. Broken arrows represents signal spreading.

The contributions of small GTPase activation to the transient and sustained phases of sLTP are distinctive. Activations of RhoA is relatively required for the transient phase of sLTP, while Cdc42 and Ras are required only for the sustained phase and Rac1 is required for both transient and sustained phases of sLTP (Harvey et al., 2008; Murakoshi et al., 2011; Oliveira and Yasuda, 2014; Hedrick et al., 2016; Figure 2B). Furthermore, they show different time courses of activity during sLTP. While activities of these GTPases remain elevated over 20 min, Rac1 displays prominently slower inactivation than those of RhoA, Cdc42 and Ras (Figure 2C). In addition, their spatial profiles are different: RhoA, Rac1 and Ras activities spread out from stimulated spine to the dendritic shaft and adjacent spines, whereas Cdc42 activity is restricted to the stimulated spine compartment (Harvey et al., 2008; Murakoshi et al., 2011; Oliveira and Yasuda, 2014; Hedrick et al., 2016; Figure 3). The spreading of Ras, RhoA and Rac1 facilitates sLTP in surrounding spines (Harvey et al., 2008; Hedrick et al., 2016; Hedrick and Yasuda, 2017). Although the regulatory mechanism of spatial constraint of Cdc42 activation is not clear, differential distributions of Cdc42 or interacting proteins such as Cdc42-specific GTPase activating protein (GAP) in dendritic shaft could limit the spreading of Cdc42 activity (Yasuda and Murakoshi, 2011; Yasuda, 2017).

In addition to these rapid actin remodeling, another major cytoskeletal protein, microtubules also may undergo large structural changes. Microtubules are formed by polymerization of tubulin monomers (Nogales et al., 1998). Usually they are localized in the dendritic shafts and excluded from dendritic spines (Korobova and Svitkina, 2010). However, microtubule tips frequently enter dendritic spines transiently, in an activity-dependent manner (Hu et al., 2008; Jaworski et al., 2009; Merriam et al., 2011). Microtubule tip entry is correlated with spine enlargement, suggesting that this phenomenon may be important for spine structural plasticity (Jaworski et al., 2009; Merriam et al., 2011, 2013). Consistent with this, microtubule dynamics has been implicated in LTP and spine structural plasticity as well as maintenance of normal spine structure (Hu et al., 2008; Jaworski et al., 2009; Merriam et al., 2011, 2013). Microtubule tip entry recruit microtubule tip binding protein EB3, which is usually used to monitor microtubule tip, and EB-binding protein p140CAP, a regulator of Src kinase activity, into spines (Jaworski et al., 2009; Merriam et al., 2013). The entry of such protein complex may play an important role in regulating actin dynamics in dendritic spines and inducing spine structural plasticity (Dent, 2017).

### Extracellular Factors Inducing Spine Remodeling

In addition to the intracellular regulation of spine structure, spine remodeling is also controlled by extracellular factors, such as autocrine signaling of brain-derived neurotrophic factor (BDNF; Harward et al., 2016; Hedrick et al., 2016) and proteolytic cleavage of extracellular matrix (ECM) and trans-synaptic cell adhesion molecules (CAMs; Sonderegger and Matsumoto-Miyai, 2014; Reinhard et al., 2015; Figure 3).

BDNF has long been deemed crucial to LTP (Minichiello, 2009). Recent studies have further demonstrated that BDNF can be released from dendritic spines during the induction of LTP to activate TrkB receptors in the same spine to regulate sLTP (Tanaka et al., 2008; Edelmann et al., 2015; Harward et al., 2016). Thus, BDNF acts as autocrine loop signaling through extracellular space of stimulated spines, which can subsequently regulate structural reorganization at the spine and adjacent spines via Rac1 and Cdc42 activities (Hedrick et al., 2016). The autocrine BDNF may play additional roles in protein synthesis (Tanaka et al., 2008), as BDNF can induce the local synthesis of several molecules including Arc, LIMK1 and CaMKIIα (Leal et al., 2014; Panja and Bramham, 2014).

Synaptic plasticity is also associated with restructuring of the extracellular space through proteolytic cleavage of the ECM and CAMs (Sonderegger and Matsumoto-Miyai, 2014; Reinhard et al., 2015). Among several endopeptidases that control synaptic functions, the matrix metalloprotease 9 (MMP-9) has been recently implicated in spine morphogenesis and synaptic plasticity (Wang X. et al., 2008; Sonderegger and Matsumoto-Miyai, 2014; Gorkiewicz et al., 2015; Magnowska et al., 2016). MMP-9 is released from dendritic spines, and cleaves components of the ECM including brevican, laminin and aggrecan, as well as CAMs such as N-cadherin and neuroligin-1 (Nagappan-Chettiar et al., 2017). In the visual cortex in adult mice, sensory experience increases MMP-9 activity, which mediates functional and morphological remodeling of synapses by digesting the ECM (Murase et al., 2017). Furthermore, NMDA receptor-mediated cleavage of intercellular adhesion molecule-5 (ICAM-5) via MMP appears to occur during LTP (Conant et al., 2010). Since ICAM-5 is known to inhibit spine maturation and enlargement by interacting with actin regulating proteins (Furutani et al., 2007), shedding of this molecule may play an important role in spine enlargement during LTP (Furutani et al., 2007; Conant et al., 2010). Interestingly, the proteolytic activity of MMP-9 is usually suppressed by tissue inhibitor of metalloproteinases-1 (TIMP-1), an endogenous inhibitor (Stawarski et al., 2014). This inhibition can be suppressed in an activity-dependent manner. Specifically, activity induces fusion of lysosomes with the plasma membrane releasing Cathepsin B extracellularly. Cathepsin B is then able to cleave TIMP-1. Thus, it appears that both MMP-9 and TIMP-1 are released from the postsynaptic cell to regulate spine structural plasticity (Padamsey et al., 2017).

In addition to the release of proteins, recent studies provide a novel concept of mRNA transmission between neurons via exosomal vesicles (EVs; Ashley et al., 2018; Pastuzyn et al., 2018). It has been known that the EV-mediated molecular transmissions occurs between neurons in an activity-dependent manner (Budnik et al., 2016). Interestingly, two studies identified that Arc protein self-assembles viral capsid, group-specific antigen (Gag)-like structure containing Arc mRNA. The EVs load this virus-like assembly and release them into extracellular space, received by other neurons (Ashley et al., 2018; Pastuzyn et al., 2018). During the transmission, mRNA is encapsulated by Arc protein and thus resistant to RNase. The received mRNA is translated, thereby producing Arc protein in the receiving neurons: even in *Arc* knockout neurons, application of the virus-like capsids can result in Arc expression (Pastuzyn et al., 2018). Since the expression of Arc, an immediate-early gene, controls functional and structural plasticity of dendritic spines (as discussed below), the EV-mediated transfer of Arc mRNA between cells might play important roles in various forms of synaptic plasticity (Pastuzyn et al., 2018).

Interestingly, Arc and viral Gag protein shares homologous DNA sequences, as well as structural similarity, indicating that *Ty3/gypsy* family of retrotransposon is presumably the ancestral origin of Arc (Campillos et al., 2006; Zhang et al., 2015). Arc and viral Gag share key functional features such as membrane binding (Barylko et al., 2018), self-oligomerization of capsid-like protein (Myrum et al., 2015; Ashley et al., 2018; Pastuzyn et al., 2018), RNA-binding and EV-mediated exosomal releases (Ashley et al., 2018; Pastuzyn et al., 2018; Shepherd, 2018). It is still elusive whether other proteins and mRNAs can be also transferred between neurons through similar mechanisms. However, human genome contains at least 103 Gag-like protein sequences including mitogen-activated protein kinase 1 (MAPK1) and retrotransposon-derived proteins PEG10 and PEG3. Interestingly, some of them are implicated in cellular development and apoptosis (Campillos et al., 2006). Therefore, these Gag-like proteins may take advantages of similar retrotransposon-like, intercellular communication systems for their physiological functions.

## Protein Synthesis-Dependent Remodeling of the Spine

Some forms of sustained structural plasticity of dendritic spines require activity-dependent protein synthesis (Tanaka et al., 2008; Govindarajan et al., 2011; Bosch et al., 2014). Growing evidence has suggested that local translation near the stimulated spines occurs, and influences cytoskeletal remodeling during spine morphogenesis (Jung et al., 2014; Rangaraju et al., 2017). We next discuss molecular networks controlling local translation and its implications for spine structural plasticity.

### Local Translations in Dendrites

**Figure 4:**
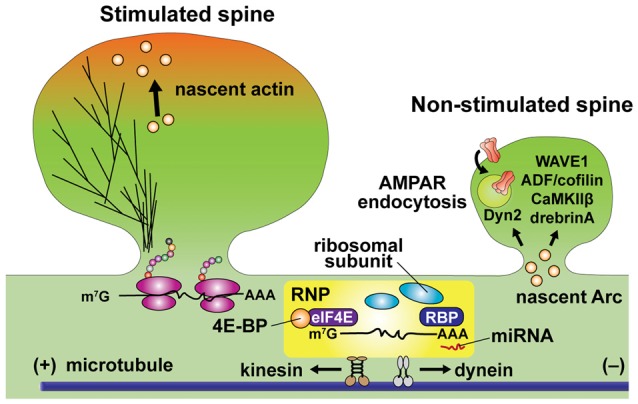
Schematic diagram of local translation in dendrites. mRNA is transported in the ribonucleoprotein (RNP) particle, which includes mRNA, translation initiation factors, ribosomal subunits, RNA binding proteins (RBPs) and microRNA (miRNA). Association with RNA binding proteins (RBPs) at 3’-UTR (representing as AAA) and eukaryotic translation initiation factor 4E (eIF4E) at 5’-cap (representing as m^7^G). RBP binding is critical for dendritic trafficking of mRNA, while repressing translation. Cap-dependent translation initiation is regulated by the interaction of mRNA with eIF4E binding protein (4E-BP). Kinesin and dynein actively transport RNP toward anterograde and retrograde directions along microtubules, respectively. Unloading of β-actin mRNA and translation initiation are simultaneously regulated at the base of stimulated spines. Newly synthesized β-actin translocates to the peripheral region of the stimulated spine head. Warm color depicts dynamic actin pools. Newly synthesized Arc preferentially increases in non-stimulated spines and changing cytoskeletal dynamics via interacting ABPs including WAVE1, ADF/cofilin, CaMKIIβ and drebrin A. Arc promotes AMPA receptor (AMPAR) endocytosis through interaction to dynamin-2 (Dyn2).

Among 2550 mRNAs localized in dendrites and axons of hippocampal neurons (Cajigas et al., 2012), many of them, such as β-actin, Arc, PSD-95, GluA1, CaMKIIα and zipcode binding protein 1 (ZBP1), are relevant to synaptic plasticity. (Mayford et al., 1996; Tiruchinapalli et al., 2003; Sutton et al., 2006; Butko et al., 2012; Steward et al., 2015; Yoon et al., 2016). In particular, dendritic localization of CaMKIIα mRNA has been suggested to be important for LTP and learning (Miller et al., 2002). Translationally inhibited mRNAs make complexes called ribonucleoprotein (RNP) particles, a transport type RNA granule (RNG) that contains mRNA, RNA binding proteins (RBPs) and microRNA (miRNA/miR; Kiebler and Bassell, 2006; Darnell, 2013; Figure 4). RNPs are actively transported by motor proteins along the cytoskeleton (Bramham and Wells, 2007). The association of trans-acting RBPs on *cis*-acting elements in 3’-untranslated region (UTR) of mRNA is critical for mRNA transport and translational repression (Kanai et al., 2004; Hüttelmaier et al., 2005; Doyle and Kiebler, 2011; Darnell and Richter, 2012). Indeed, CaMKIIα mRNA lacking the 3’-UTR is not trafficked to the dendrite and impairs late-LTP and learning (Miller et al., 2002). “Unloading” of mRNA at the correct location is regulated by RBP phosphorylation, which causes mRNA dissociation and initiates the translation (Fernandez-Moya et al., 2014). In addition, inhibition of cap-dependent translation initiation during consolidation of aversive conditioning impairs the accumulation of polyribosomes in spines in the hippocampus (Ostroff et al., 2017). Thus, mRNA transport and cap-dependent translation may coincide specifically at activated spines during memory consolidation.

Recent advances in single mRNA imaging techniques have revealed a number of fine details about RNP transport in dendrites (Glock et al., 2017). Single-molecule fluorescence *in situ* hybridization (smFISH) revealed that, in dendrites, β-actin mRNA in RNA granules is sterically masked from the translation machinery as well as FISH probes, and unmasked by neuronal activity (Buxbaum et al., 2014). Live-cell imaging of RNA granules also became possible using a knock-in mouse line in which multiple MS2 binding site (MBS) stem loops are inserted at 3’-UTR of β-actin mRNA. By expressing MS2-GFP in these mice, endogenous mRNA can be fluorescently labeled. This technique has revealed that many of the RNPs in dendrites move in a bidirectional manner (Lionnet et al., 2011; Yoon et al., 2016), indicating that RNPs are transported along microtubules by both kinesin and dynein molecular motors (Kanai et al., 2004; Gagnon and Mowry, 2011). When sLTP is induced at a single spine with glutamate uncaging, mobile mRNA particles are stopped at the base of the stimulated spine, and newly synthesized actin appears at the tip of the stimulated spine (Buxbaum et al., 2015; Yoon et al., 2016). These events may correspond to mRNA unloading and translation near or in the stimulated spine by activity-dependent phosphorylation of RBPs in dendrites (Rangaraju et al., 2017).

Recently techniques to simultaneously monitor single mRNAs and newly translated polypeptides in intact cells have been developed (Chekulaeva and Landthaler, 2016; Morisaki et al., 2016; Wang et al., 2016; Wu et al., 2016; Yan et al., 2016). These techniques may further improve our knowledge about the regulation of local dendritic translation required for morphogenesis of dendritic spines.

### Interactions Between Regulators of Protein Synthesis and That of Actin Cytoskeleton

The finding of activity-dependent β-actin translation near stimulated spines provides a direct link between protein synthesis and actin dynamics during spine morphogenesis (Yoon et al., 2016). Recent studies also suggest more intricate interactions between cytoskeletal proteins and synthesized proteins. One form of interaction is through the synthesis of actin regulating proteins. For example, it has been reported that oligophrenin-1 (OPHN1), a Rho GTPase activating protein (Rho-GAP), is rapidly upregulated by activity, regulating actin cytoskeleton during mGluR-dependent LTD (Nadif Kasri et al., 2011). In addition, RhoA appears to be locally synthesized in response to BDNF application or neuronal activity, and plays an important role in LTP (Briz et al., 2015). Interestingly, local translation of Arc, an activity-dependent immediate early gene, is also reported to regulate actin cytoskeleton and control spine morphology during LTP and LTD (Newpher et al., 2018). It has been known that newly synthesized Arc can interact with actin regulatory proteins such as WAVE1, ADF/cofilin, CaMKIIβ and drebrin A, regulating actin filaments (Messaoudi et al., 2007; Okuno et al., 2012; Zhang et al., 2015; Nair et al., 2017; Figure 4). Arc is also known to interact with dynamin-2 to promote endocytosis of AMPA receptor (AMPARs; Chowdhury et al., 2006; Newpher et al., 2018). The location of Arc synthesis and its regulation of actin cytoskeleton has been also studied extensively. It has been suggested that newly synthesized Arc is accumulated in non-stimulated spines and suppresses synaptic potentiation by decreasing surface expression of AMPA receptors (Okuno et al., 2012). This is in general consistent with the role of Arc expression in inhibiting LTP (Plath et al., 2006; Rial Verde et al., 2006).

**Figure 5:**
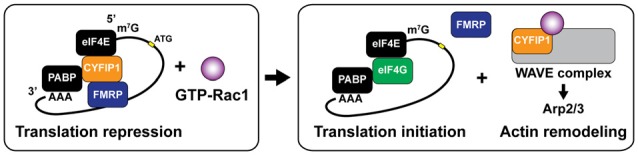
Dual roles ofcytoplasmic fragile X mental retardation protein (FMRP) interacting protein 1 (CYFIP1) for translation initiation and rapid actin remodeling. CYFIP1 makes FMRP-CYFIP1-eIF4E complex on mRNA and plays a role as a non-canonical eukaryotic initiation factor 4E (eIF4E) binding protein (4E-BP) which represses the translation initiation by interfering the association of eukaryotic initiation factor 4G (eIF4G). Binding of active form Rac1 (GTP-Rac1) dissociates CYFIP1 from the mRNA complex and initiates the translation. Dissociated CYFIP1 from mRNA complex forms WAVE complex, which promotes actin nucleation and branching via activating Arp2/3 complex. PABP, poly (A)-binding protein.

Another interesting interaction between local translation and cytoskeletal remodeling involves the dual roles of cytoplasmic fragile X mental retardation protein (FMRP) interacting protein 1 (CYFIP1, Sra1). It has been demonstrated that CYFIP1 regulates spine remodeling both through promoting translation initiation and Arp2/3-mediated rapid actin nucleation (De Rubeis et al., 2013; Figure 5). CYFIP1 has been identified as a non-canonical eukaryotic translation initiation factor 4E (eIF4E) binding protein (4E-BP), which repress cap-dependent translation by forming a complex with FMRP and eIF4E (Napoli et al., 2008; Udagawa et al., 2012; Panja and Bramham, 2014). BDNF stimulation induces the dissociation of CYFIP1 from the FMRP-CYFIP1-eIF4E complex at synapses, which upregulates translation initiation of Arc, MAP1B and CaMKIIα (Napoli et al., 2008; De Rubeis and Bagni, 2011). The release of CYFIP1 occurs through the activity of Mnk1, a downstream molecule of MAPK/ERK signaling (Genheden et al., 2015; Bramham et al., 2016). In addition to the induction of translation initiation, the dissociated CYFIP1 can form a WAVE regulatory complex (WRC) that triggers Arp2/3-dependent actin nucleation with interacting Rac1 (Derivery et al., 2009; De Rubeis and Bagni, 2011). Indeed, either knockdown of *Cyfip1* or mutations of interacting regions with eIF4E or WRC impairs structural maturation of dendritic spines (De Rubeis et al., 2013), suggesting that interplay of CYFIP1 controls both translation-dependent and -independent remodeling of the spine structure.

Finally, the mTOR complex 2 (mTORC2) pathway, a major pathway that regulates protein synthesis, is also found to regulate dynamics of actin cytoskeleton through Rac1 activation (Huang et al., 2013; Switon et al., 2017). Although the precise mechanism of mTORC2-mediated remodeling of actin cytoskeleton remains elusive, it has been proposed that the recruitment of Tiam1, a Rac1-specific guanine exchange factor (GEF), by Rictor, an essential component of mTORC2, regulates Rac1 activity and subsequent actin polymerization (Huang et al., 2013).

### Local Translational Repression and Degradation

miRNAs are short (21–25 nucleotides) non-coding RNAs, which have important roles for specific translational control at synapses. miRNAs silence translation and induce degradation via making RNA-induced silencing complex (RISC) and binding corresponding sequences in 3’-UTR of mRNAs (Wahid et al., 2010). Since miRNAs have moderate specificity due to their short sequences, they can target a group of mRNAs. Many miRNA have been identified as regulators of actin related proteins and synaptic plasticity (Ryan et al., 2015). For example, miR-134 makes RISC with association of Argonaute (Ago) protein and represses translation of LIMK1 in dendritic spines of hippocampal neurons by NMDAR-dependent manner, decreasing the size of dendritic spines (Schratt et al., 2006; Rajgor et al., 2018). miR-132 is upregulated in an activity-dependent manner through the MAPK/ERK pathway, and represses translation of p250GAP, a brain-enriched Rho-GAP (Wayman et al., 2008). p250GAP is known to regulate spine morphology and is implicated in wide range of neuropathologies (Nakazawa et al., 2008; Impey et al., 2010; Qian et al., 2017). In dendrites, miR-138 represses acyl-protein thioesterase 1 (APT1), a depalmitoylation enzyme, and thus inhibits spine growth via increasing membrane localization of α_13_ subunits of G proteins (Gα13), which upregulates RhoA activity and presumably promotes actin depolymerization (Siegel et al., 2009).

Interestingly, miRNA maturation process appears to be localized near and in stimulated spines (Sambandan et al., 2017). The study by Sambandan et al. (2017) used a cleavage-inducible fluorescent sensor to measure the activity of Dicer and demonstrated that the maturation of miR-181a occurs in spines stimulated with glutamate uncaging. Thus, miRNAs may act locally to regulate activity-dependent tuning of the translation in stimulated spines. Moreover, the authors visualized newly synthesized CaMKIIα by using proximity ligation assay (PLA)-based technique (tom Dieck et al., 2015) and found that nascent CaMKIIα showed marked reduction in the area generated the mature miRNA (Sambandan et al., 2017). Thus, precise spatiotemporal maturation of miRNA regulates local translation of key signaling molecules in dendritic subcompartments. Taken together with the important role of miRNAs in regulating the actin cytoskeleton, miRNA-mediated translation repression and degradation may be a key regulatory system for structural plasticity of dendritic spines.

## Concluding Remarks

Structural plasticity of dendritic spines is regulated by the reorganization of actin cytoskeleton through interaction with ABPs and their regulatory molecules. Ca^2+^ elevation in spines activates multiple signaling pathways that relay short Ca^2+^ signals into much longer signals. These signaling pathways have specific spatiotemporal patterns that orchestrate different aspect of dynamic cytoskeletal regulation, such as polymerization, depolymerization, nucleation, cross-linking and capping in the stimulated spines. In addition, activity-dependent signaling such as RNA trafficking and miRNAs regulate local protein translation of β-actin and actin regulatory proteins, providing an efficient local supply of the necessary material (Holt and Schuman, 2013; Rangaraju et al., 2017). The regulation of cytoskeletal elements and translational regulation appear to be coupled, providing an additional layer of regulation to structural plasticity (De Rubeis et al., 2013; Buxbaum et al., 2015; Yoon et al., 2016; Rangaraju et al., 2017; Sambandan et al., 2017).

Many neurological disorders, including autism spectrum disorders (ASDs), schizophrenia and Fragile X syndrome (FXS), are thought to be associated with dysregulation of cytoskeletal and translational signals (Buffington et al., 2014; Huber et al., 2015; Bhambhvani et al., 2017; Joensuu et al., 2018). These two signaling systems appear to be tightly coupled and important for regulating spine structural plasticity (De Rubeis et al., 2013; Hadziselimovic et al., 2014). Further studies of the interplay between the local regulation of cytoskeleton and translation during spine structural plasticity will be provide us with a better understanding of these diseases as well as basic understanding of spine structural plasticity.

Signal transduction regulating structure and function of dendritic spines are exceedingly complicated, and it will be long way to understand the whole signaling system. Due to the morphological complexity of neurons, the spatiotemporal dynamics of signaling play particularly important roles in neuronal plasticity. While quantitative measurements of more detailed signaling pathways will lead to a better understanding of overall signaling system, it would be critical to create theoretical frameworks that can integrate the spatiotemporal dynamics of many different signaling pathways (Brown et al., 2011). As we observe more sub-spine signal compartmentalization, the theory needs to include sub-spine structure and compartmentalization (Colgan and Yasuda, 2014). Finally, since most of our efforts of measuring signal transduction is still limited to *in vitro* models such as cultured neurons and brain slices, developments of systems to measure signaling activity in live animals with high spatiotemporal resolution would be necessary to connect biochemical events single dendrite with neuronal circuit plasticity in specific behavioral paradigm.

## Author Contributions

Both authors listed have made a substantial, direct and intellectual contribution to the work, and approved it for publication.

## Conflict of Interest Statement

The authors declare that the research was conducted in the absence of any commercial or financial relationships that could be construed as a potential conflict of interest.
